# *Rickettsia parkeri* Infection after Tick Bite, Virginia

**DOI:** 10.3201/eid1302.061295

**Published:** 2007-02

**Authors:** Timothy J. Whitman, Allen L. Richards, Christopher D. Paddock, Cindy L. Tamminga, Patrick J. Sniezek, Ju Jiang, David K. Byers, John W. Sanders

**Affiliations:** * National Naval Medical Center, Bethesda, Maryland, USA; † Naval Medial Research Center, Silver Spring, Maryland, USA; ‡ Centers for Disease Control and Prevention, Atlanta, Georgia, USA

**Keywords:** Rickettsia parkeri, rickettsia, Amblyomma, tick, military, dispatch

## Abstract

We describe a man with a febrile illness and an eschar that developed at the site of a tick bite. *Rickettsia parkeri* was detected and isolated from the eschar. This report represents the second documented case of *R. parkeri* rickettsiosis in a US serviceman in eastern Virginia.

In the United States, 4 species of spotted fever group (SFG) rickettsiae are recognized as pathogens of humans. These include *Rickettsia rickettsii*, the cause of Rocky Mountain spotted fever (RMSF); *R. felis*, the cause of fleaborne spotted fever; *R. akari*, the agent of rickettsialpox; and *R. parkeri* (*1*,*2*). Of these, *R. rickettsii* is the only pathogen definitely associated with tick bites.

In 2004, Paddock et al. described the first recognized case of infection in a patient with *R. parkeri (1).* That patient, a US serviceman living in the Tidewater region of eastern Virginia, had a mild febrile illness and multiple eschars. He reported frequent tick and flea exposures but could not recall a specific arthropod bite in the month before illness. However, *R. parkeri,* a tick-associated rickettsia (*3*), was subsequently isolated in cell culture from 1 eschar. (*1*) We present the second known case of spotted fever due to *R*. *parkeri* in a serviceman (the third case overall) and its unequivocal association with tick bite.

## The Case

A 53-year-old US serviceman was seen at our clinic on September 8, 2006; he reported a 2-day history of fever, malaise, and rash. He denied headache, nausea, vomiting, or myalgia. He had recently returned from a vacation in the Virginia Beach area, where he had removed a large brown tick with white markings from his right pretibial region. The patient estimated that the tick had been attached ≈8 hours before being removed. Four days after the tick was removed, an eschar developed at the bite site. Three days later, temperatures up to 39°C and drenching night sweats that persisted for 2 days developed. He then sought care.

**Figure 1:**
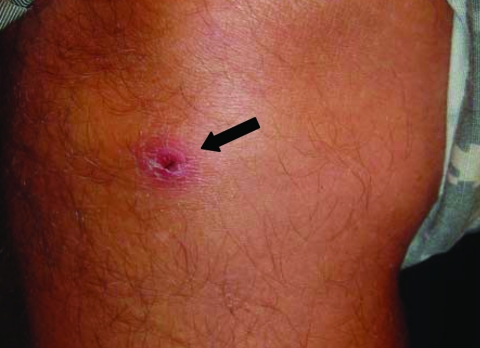
Eschar on right pretibial region.

**Figure 2:**
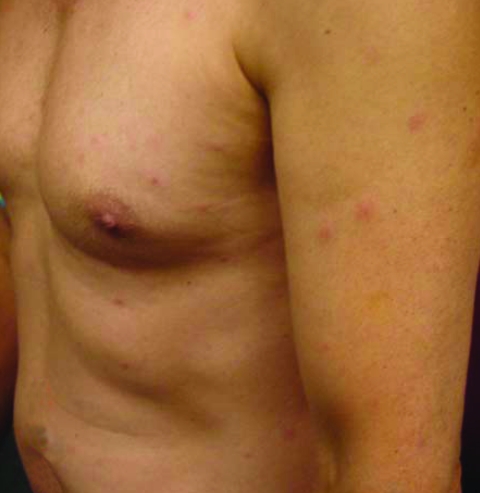
Multiple papules on torso, upper arms, and legs.

On examination, his temperature was 38°C. A nontender, nonpruritic eschar that measured 1 cm × 1 cm was visible on his right lower extremity (Figure 1) along with ≈15 nontender, nonpruritic papules on his torso, upper arms, and legs (Figure 2). The remainder of his physical examination was normal. Laboratory studies were normal except for a leukocyte count of 3.4 × 10^9^ cells/L (normal 4–11 × 10^9^ cells/L) and an aspartate aminotransferase level of 38 U/L (normal 8–33 U/L).

The patient was admitted to the hospital and treated with oral doxycycline 100 mg twice a day. Fevers and malaise immediately resolved after the first dose. The patient was discharged, and the rash resolved after 3–4 days of therapy.

DNA was extracted from skin-biopsy specimens of the proximal tibial eschar and a shoulder papule and evaluated by using 2 real-time PCR assays designed to amplify segments of the 17-kDa antigen and outer membrane protein B genes of all *Rickettsia* spp. and tickborne SFG rickettsiae, respectively *(**4**,**5**)*. Amplicons obtained from 2 additional genes, *gltA* (333 bp) and *sca4* (849 bp), were sequenced and determined to be 100% identical to those of *R. parkeri* (GenInfo Identifier nos. 1389996; 13568656). The histopathology of the shoulder papule showed mild superficial perivascular inflammation without eosinophils. Acute-phase serum and whole blood samples from the patient were also tested by real-time PCR and were negative. *R. parkeri* (Ft. Story strain) was isolated in Vero E6 cells from a portion of the eschar biopsy specimen. Immunohistochemical staining of the shoulder papule identified occasional spotted fever rickettsiae in the inflammatory cell infiltrate. Acute- and convalescent-phase serum specimens were tested for immunoglobulin G antibodies reactive with SFG and typhus group (TG) rickettsiae by using ELISAs with *R. rickettsii* and *R. typhi* antigens. The titers for the SFG ELISA acute- and convalescent-phase serum samples were <100 and 1,600, respectively. Both samples were negative (titer <100) for the TG ELISA.

## Conclusions

*R. parkeri* was first isolated in 1937 from *Amyblyomma maculatum* ticks from cattle in the Gulf Coast region of Texas (*3*), but its role as a human pathogen was unknown. For many years investigators speculated that agents other than *R. rickettsii*, including *R. parkeri*, caused mild RMSF-like illnesses in the United States (*6*,*7*). However, the role of *R. parkeri* as a pathogen of humans was not confirmed until 2002, when *R. parkeri* was isolated from a patient with a relatively mild febrile illness and multiple eschars (*1*). Confirmed cases of *R. parkeri* rickettsiosis have been described only twice (*1*,*8*), and the prevalence of this disease is unknown; however, many cases of this infection have likely been misidentified as RMSF (*6*). Recently, Raoult and Paddock analyzed serum specimens of 15 US patients who had an earlier diagnosis of RMSF and identified 4 that reacted with a 120-kDa protein of *R. parkeri*, a finding suggestive of infection with this agent (*9*). This hypothesis is further supported by seroprevalence studies of US military personnel that showed that 10% those tested were positive for SFG rickettsiae when a *R. rickettsii* ELISA antigen was used (*10*). Because of recognized cross-reactivity of *R. rickettsii* with antigens of other SFG rickettsiae, including *R. parkeri*, this level of serologic reactivity could represent mild, self-limited infections with other SFG rickettsiae, including *R. parkeri,* that are less virulent than *R. rickettsii*.

Our patient had a single eschar at the site of tick attachment. Although it has been presumed that ticks are involved in the transmission of this disease, this is the first clear documentation of this occurrence. The infection can apparently be transmitted within several hours of attachment because the patient was certain of the maximum interval that the tick could have been attached to a visible region of his leg. Finally, the general description of the tick matches that of *A. maculatum*, the putative vector of *R. parkeri* rickettsiosis, and this tick has been collected previously in southeastern Virginia (*11*).

*R. parkeri* is the newest member of the SFG rickettsiae in the Western Hemisphere to be conclusively associated with illness in humans (*12*). Because 2 of the 3 patients described to date were US servicemen in the Virginia Tidewater region, where thousands of military personnel are stationed, this tickborne infection has potential military relevance. Serologic tests specifically for *R. parkeri* are not widely available, and an accurate diagnosis is best made by using PCR testing of a biopsy specimen of eschars or papules. A definitive diagnosis can also be made by using cell culture isolation techniques with the same biopsy specimen, as described for other eschar-associated rickettsioses (*12*).

Clinicians should be aware of this newly noted rickettsial illness, specifically in the eastern coastal region of the United States. They should consider this diagnosis for patients with mild rickettsiosis-like illness and single or multiple eschars.
